# Impact of intensive care unit admission during morning bedside rounds and mortality: a multi-center retrospective cohort study

**DOI:** 10.1186/cc11329

**Published:** 2012-05-03

**Authors:** Ivens Augusto O de Souza, Constantine J Karvellas, RT Noel Gibney, Sean M Bagshaw

**Affiliations:** 1 Adult Intensive Care Unit, Hospital Sirio-Libanês, Rua Dona Adma Jafet, 91 - Bela Vista, CEP: 01308-050, Sao Paulo/SP, Brazil; 2 Division of Critical Care Medicine, Faculty of Medicine and Dentistry, University of Alberta, 3C1.12 Walter C. Mackenzie Centre, 8440-122 Street, Edmonton, AB, T6G 2B7, Canada

## Abstract

**Introduction:**

Recent data have suggested that patient admission during intensive care unit (ICU) morning bedside rounds is associated with less favorable outcome. We undertook the present study to explore the association between morning round-time ICU admissions and hospital mortality in a large Canadian health region.

**Methods:**

A multi-center retrospective cohort study was performed at five hospitals in Edmonton, Canada, between July 2002 and December 2009. Round-time ICU admission was defined as occurring between 8 and 11:59 a.m. Multivariable logistic regression analysis was used to explore the association between round-time admission and outcome.

**Results:**

Of 18,857 unique ICU admissions, 2,055 (10.9%) occurred during round time. Round-time admissions were more frequent in community hospitals compared with tertiary hospitals (12.0% vs. 10.5%; odds ratio [OR] 1.16; 95% CI, 1.05-1.29, *P *< 0.004) and from the ward compared with the emergency department (ED) or operating theater (17.5% vs. 9.2%; OR 2.1; 95% CI, 1.9-2.3, *P *< 0.0001). Round-time admissions were more often medical than surgical (12.6% vs. 6.6%; OR 2.06; 95% CI, 1.83-2.31, *P *< 0.0001), had more comorbid illness (11.9% vs. 10.5%; OR 1.15; 95% CI, 1.04-1.27, *P *< 0.008) and higher APACHE II score (22.2 vs. 21.3, *P *< 0.001), and were more likely to have a primary diagnosis of respiratory failure (37.0% vs. 31.3%, *P *< 0.001) or sepsis (11.1% vs. 9.0%, *P *= 0.002). Crude ICU mortality (15.3% vs. 11.6%; OR 1.38; 95% CI, 1.21-1.57, *P *< 0.0001) and hospital mortality (23.9% vs. 20.6%; OR 1.21; 95% CI, 1.09-1.35, *P *< 0.001) were higher for round-time compared with non-round-time admissions. In multi-variable analysis, round-time admission was associated with increased ICU mortality (OR 1.19, 95% CI, 1.03-1.38, *P *= 0.017) but was not significantly associated with hospital mortality (OR 1.02; 95% CI, 0.90-1.16, *P *= 0.700). In the subgroup admitted from the ED, round-time admission showed significantly higher ICU mortality (OR 1.54; 95% CI, 1.21-1.95; *P *< 0.001) and a trend for higher hospital mortality (OR 1.22; 95% CI, 0.99-1.51, *P *= 0.057).

**Conclusions:**

Approximately 1 in 10 patients is admitted during morning rounds. These patients are more commonly admitted from the ward and are burdened by comorbidities, are non-operative, and have higher illness severity. These patients admitted during morning rounds have higher observed ICU mortality but no difference in hospital mortality.

## Introduction

Bedside clinical rounds are an integral component for both patient care and medical education. Intensivists in academic medical centers and many metropolitan/community-based hospitals often must balance evidence-based and high-quality patient care with appropriate supervision of trainees, including medical students, residents, and fellows.

The acuity of illness in the first few hours after patient admission to the intensive care unit (ICU) is most often used to prognosticate the clinical course on the basis of the premise that these patients are most physiologically unstable during this period [1]. This represents a crucial phase for resuscitation, often requiring active and diligent bedside multi-disciplinary care. The concept of early, prompt, and aggressive intervention to reverse physiologic instability has been shown to improve outcomes across a range of critically ill states, including sepsis [2], ischemic stroke [3], cardiac arrest [4,5], and acute myocardial infarction [6]. However, in the morning, the ICU team is often focused on multi-disciplinary bedside clinical and teaching rounds. New ICU admissions occurring during this period may disrupt workflow and, in selected circumstances, negatively impact the care of other ICU patients. Likewise, patients admitted during morning rounds may have their transfer to the ICU or the initiation of timely diagnostic, supportive, and therapeutic measures (or both) delayed [7].

Recently, in a retrospective cohort study, Afessa and colleagues [8] found an increase in mortality for critically ill patients admitted to the ICU during morning bedside clinical rounds. These data imply that system-level factors, such as hospital/ICU organizational structure, have the potential to negatively impact patient outcome. This retrospective study, however, was performed at a single academic institution and may not be widely generalizable.

Accordingly, to further explore the hypothesis that ICU admission during morning rounds portends risk of worse clinical outcome, we performed a multi-center cohort study at five teaching hospitals in a large Canadian health region. The objective was to investigate the effect of round-time ICU admissions on ICU and hospital mortality in critically ill patients.

## Materials and methods

The study was approved by the Health Research Ethics Board at the University of Alberta prior to commencement. The requirement for individual informed consent was waived. The reporting of this study follows the STROBE (strengthening the reporting of observational studies in epidemiology) guideline [9].

### Study design and setting

This was a multi-center retrospective observational cohort study performed at five teaching-affiliated hospitals in Edmonton, Canada, between 1 July 2002 and 31 December 2009. Edmonton has an estimated population of 730,000 and a metropolitan population of 1,034,945. The three metropolitan/community hospitals are the Grey Nuns Community Hospital (GNH) (eight beds and one daytime/one night-time intensivist), Misericordia Community Hospital (six beds and one daytime/one night-time intensivist), and Sturgeon General Hospital (six beds and one daytime/one night-time intensivist). These three hospitals all have mixed medical/surgical ICUs. The GNH is the regional referral center for all major vascular surgery. The two tertiary hospitals are the Royal Alexandria Hospital (RAH) (22 beds and two daytime intensivist teams and one night-time intensivist) and University of Alberta Hospital (UAH) (30 beds and three daytime intensivist teams and one night-time intensivist). Both of these tertiary hospitals have mixed medical/surgical/trauma ICUs. The RAH is a level II trauma center, and the UAH is a level I trauma center and the regional referral center for all non-cardiac solid-organ transplantation. Bedside morning rounds in each ICU were attended to by one or more consultant intensivists, residents, and/or bedside clinicians and by a multi-disciplinary team consisting of registered nurses, respiratory therapists, pharmacists, dieticians, and physiotherapists. Critical care fellows rotate between the ICUs, and the majority of their rotations occur at the two tertiary hospital sites.

### Study population

All consecutive adult (at least 18 years old) patients admitted to one of the five hospitals' participating ICUs were eligible. If a patient had multiple ICU admissions during a single hospitalization, only the first admission was included for analysis. Patients discharged from the ICU less than 24 hours after admission and those with missing data on vital status were excluded.

### Study definitions

Round-time admission was defined as an ICU admission occurring between 8 and 11:59 a.m., corresponding to the routine time during which multi-disciplinary and teaching bedside clinical rounds are performed at all participating ICUs. Non-round-time admission was defined as an ICU admission occurring between noon and 7:59 a.m. ICU admission source was classified as emergency department (ED), operating room/emergency post-operative status, operating room/elective post-operative status, transfer from other institutions, or in-hospital ward transfer. Severity of illness was defined according to the Acute Physiology and Chronic Health Evaluation II (APACHE II) score [10]. Hepatic failure was defined as having documented cirrhosis by histology or elevated bilirubin and international normalized ratio attributed to liver disease. Immunosuppression was defined as having received cytotoxic medication or steroids or both within the 7 days preceding ICU admission. Chronic respiratory disease was defined as a documented need for home oxygen therapy or severe exercise restriction or both. Chronic kidney disease was defined as chronic dialysis therapy. Hematologic cancer was defined as having pathologically confirmed lymphoma, leukemia, or multiple myeloma. Congestive heart failure was defined as having symptoms at minimal exertion. Surgical status was defined as having had an operative procedure within 7 days of ICU admission.

### Data sources

We used an ICU-specific clinical/administrative database maintained by the regional Division of Critical Care Medicine, termed the Minimal Data Set database, which routinely captures demographic, diagnostic, clinical, physiologic, and outcome data for all ICU admissions to the five participating hospitals. We extracted data on demographics, ICU admission source/time, post-operative status, comorbidities, admission diagnoses, necessity for invasive mechanical ventilation, APACHE II score, ICU and hospital duration of stay, and ICU and hospital mortality.

### Statistical analysis

The primary exposure of interest was time of ICU admission (round-time versus non-round-time). The primary outcome measure was hospital mortality. The secondary outcome measures were ICU mortality and ICU and hospital lengths of stay. Continuous normally or near-normally distributed data are reported as means with standard deviations and compared by Student *t *test. Non-normally distributed continuous data are reported as medians and interquartile ranges and were compared by Mann-Whitney *U *test. Categorical variables were compared by using the chi-squared test. Separate customized multi-variable logistic regression models with ICU or hospital mortality as dependent variable and round-time admission as an independent variable were created and were adjusted for demographics, comorbidity, APACHE II score, use of mechanical ventilation, surgical status, admission source, and primary diagnostic category, study year, and center. Data are reported as odds ratios (ORs) with 95% confidence intervals (CIs). Data were evaluated for multi-colinearity. Model calibration and fit were assessed by the area under the receiver operating characteristic curve (AUR ROC) and the Pearson goodness-of-fit (GoF) test, respectively. All statistical analyses were two-sided, and a *P *value of less than 0.05 was considered significant. Statistical analyses were conducted by using Intercooled Stata Release 10 (StataCorp LP, College Station, TX, USA).

## Results

There were 24,829 ICU admissions during the study period. After exclusion of 1,732 (7.0%) because of repeat admissions, 3,841 (15.5%) for an ICU stay of less than 24 hours, and 399 (1.6%) because of missing data on vital status, the study cohort consisted of 18,857 unique ICU admissions (75.9%). Of these, 2,055 (10.9%) occurred during round time, from 8 to 11:59 a.m., and 16,802 (89.1%) during non-round time, from noon to 7:59 p.m. The two tertiary hospitals accounted for the majority of admissions (73.4%) during the study period (Table 1). However, round-time admissions were more common in the community ICUs when compared with the tertiary ICUs (12.0% versus 10.5%, OR 1.16, 95% CI 1.05 to 1.29, *P *= 0.004).

**Table 1:** Distribution of intensive care unit admissions among the five hospital sites, stratified by time of the admission

Hospitals	Total admissions n = 18,857	Round-time admissions **8-11:59 a.m**. n = 2,055 (10.9%)	Non-round-time admissions **Noon-7:59 a.m**. n = 16,802 (89.1%)
Community 1	2,472	258 (10.4)	2,214 (89.6)
Community 2	1,815	249 (13.7)	1,566 (86.3)
Community 3	731	95 (13.0)	636 (87.0)
Total (Community)	5,018 (26.6)	602 (12.0)	4,416 (88.0)
Tertiary 1	6,286	562 (8.9)	5,724 (91.1)
Tertiary 2	7,553	891 (11.8)	6,662 (88.2)
Total (Tertiary)	13,839 (73.4)	1,453 (10.5)	12,386 (89.5)

Values are presented as number (percentage).

### Patient characteristics

Patients admitted at round time were more likely to be admitted from the hospital ward when compared with the ED or operating theater (17.5% versus 9.2%, OR 2.10, 95% CI 1.90 to 2.30, *P *< 0.0001) (Table 2). Round-time admissions were more often medical than surgical (12.6% versus 6.6%, OR 2.06, 95% CI 1.83 to 2.31, *P *< 0.0001). Round-time patients also had greater comorbid illness (11.9% versus 10.5%, OR 1.15, 95% CI 1.04 to 1.27, *P *< 0.008), and those with any comorbidity were more likely to have a higher number of conditions (4 versus 3, *P *= 0.01). Specifically, round-time patients had higher prevalences of hematologic malignancies (*P *= 0.005), chronic kidney disease (*P *= 0.04), and immunosuppression (*P *= 0.002) when compared with non-round-time admissions. Acuity of illness was higher for round-time admissions compared with non-round-time (APACHE II score of 22.2 versus 21.3, *P *< 0.001). Round-time admissions were more likely to have primary diagnoses of respiratory failure (37.0% versus 31.3%, *P *< 0.001) and sepsis (11.1% versus 9.0%, *P *= 0.002) and were less likely to have diagnoses of cardiovascular (11.5% versus 13.8%, *P *= 0.004) or gastrointestinal (12.1% versus 17.6%, *P *< 0.001) failure in comparison with non-round-time admissions, respectively.

**Table 2:** Baseline characteristics of the study cohort, stratified by time of intensive care unit admission

Characteristics	Total admissions n = 18,857	Round-time admissions n = 2,055 (10.9%)	Non-round-time admissions n = 16,802 (89.1%)	*P *value
Male sex, number (percentage)	10,884 (57.7)	1,205 (58.6)	9,679 (57.6)	0.372
Age in years, mean (SD)	58.1 (17.8)	57.6 (18.0)	58.2 (17.8)	0.177
APACHE II score, mean (SD)	21.4 (8.0)	22.2 (8.4)	21.3 (8.0)	< 0.001
Medical admission, number (percentage)	13,491 (71.5)	1,703 (82.8)	11,788 (70.2)	< 0.001
Post-operative, number (percentage)	5,366 (28.5)	352 (17.1)	5,014 (29.8)	< 0.001
Comorbid illness, number (percentage)				
None	14,162 (75.1)	1,494 (72.7)	12,668 (75.4)	0.008
One	4,109 (21.8)	478 (23.3)	3,631 (21.6)	0.087
Two or more	586 (3.1)	83 (4.0)	503 (3.0)	0.010
Comorbid condition, number (percentage)				
Hepatic failure	967 (5.1)	98 (4.8)	869 (5.2)	0.434
Immunosuppression	1,079 (5.7)	148 (7.2)	931 (5.5)	0.002
Chronic lung disease	1,435 (7.6)	177 (8.6)	1,258 (7.5)	0.069
Chronic renal failure	592 (3.1)	80 (3.9)	512 (3.1)	0.038
Hematologic cancer	369 (1.9)	57 (2.8)	312 (1.9)	0.005
Metastatic cancer	463 (2.5)	51 (2.5)	412 (2.5)	0.935
Congestive heart failure	307 (1.6)	30 (1.5)	277 (1.7)	0.523
AIDS	90 (0.5)	9 (0.4)	81 (0.5)	0.784
Routine cardiac surgery	14 (0.1)	2 (0.1)	12 (0.1)	0.684
Primary diagnosis, number (percentage)				
Respiratory	6,020 (31.9)	760 (37.0)	5,260 (31.3)	< 0.001
Gastrointestinal	3,207 (17.0)	248 (12.1)	2,959 (17.6)	< 0.001
Cardiovascular	2,555 (13.6)	236 (11.5)	2,319 (13.8)	0.004
Sepsis	1,738 (9.2)	228 (11.1)	1,510 (9.0)	0.002
Trauma	1,658 (8.8)	180 (8.8)	1,478 (8.8)	0.955
Metabolic	1,333 (7.1)	148 (7.2)	1,185 (7.1)	0.803
Neurologic	1,316 (7.0)	146 (7.1)	1,170 (7.0)	0.813
Renal	551 (2.9)	60 (2.9)	491 (2.9)	0.995
Other	479 (2.5)	49 (2.4)	430 (2.6)	0.635
Mechanical ventilation, number (percentage)	15,076 (79.9)	16,439 (79.8)	13,437 (80.0)	0.817
Source of admission, number (percentage)				
Operating room - Elective	2,221 (11.8)	130 (6.3)	2,091 (12.4)	< 0.001
Operating room - Emergency	3,145 (16.7)	222 (10.8)	2,923 (17.4)	< 0.001
Emergency department	6,506 (34.5)	719 (35.0)	5,787 (34.4)	0.623
Other hospital	3,212 (17.0)	320 (15.6)	2,892 (17.2)	0.062
Ward	3,773 (20.0)	664 (32.3)	3,109 (18.5)	< 0.001

APACHE II, Acute Physiology and Chronic Health Evaluation II; SD, standard deviation.

### Clinical outcomes

Crude ICU mortality (15.3% versus 11.6%, OR 1.38, 95% CI 1.21 to 1.57, *P *< 0.0001) and hospital mortality (23.9% versus 20.6%, OR 1.21, 95% CI 1.09 to 1.35, *P *< 0.001) were significantly greater for ICU admissions occurring during round time compared with non-round time (Table 3). There were no significant differences in ICU or hospital lengths of stay between round-time and non-round-time admissions.

**Table 3:** Mortality and lengths of stay stratified by intensive care unit admission time

Outcomes	Total admissions n = 18,857	Round-time admissions n = 2,055 (10.9%)	Non-round-time admissions n = 16,802 (89.1%)	*P *value
Hospital mortality, number (percentage)				
All	3,946 (20.9)	491 (23.9)	3,455 (20.6)	< 0.001
Operating room - Elective	176 (7.9)	7 (5.4)	169 (8.1)	0.269
Operating room - Emergency	532 (16.9)	36 (16.2)	496 (17.0)	0.773
ED admission	1,418 (21.8)	176 (24.5)	1,242 (21.5)	0.065
Other hospital admission	595 (18.5)	61 (19.1)	534 (18.5)	0.794
Ward admission	1,225 (32.5)	211 (31.8)	1,014 (32.6)	0.676
Community hospitals admissions	1,049 (20.9)	148 (24.6)	901 (20.4)	0.018
Tertiary hospitals admissions	2,897 (20.9)	343 (23.6)	2,554 (20.6)	0.008
ICU mortality, number (percentage)				
All	2,262 (12.0)	315 (15.3)	1,947 (11.6)	< 0.001
Operating room - Elective	56 (2.5)	1 (0.8)	55 (2.6)	0.189
Operating room - Emergency	272 (8.6)	22 (9.9)	250 (8.5)	0.488
ED admission	854 (13.1)	125 (17.4)	729 (12.6)	< 0.001
Other hospital admission	415 (12.9)	47 (14.7)	368 (12.7)	0.321
Ward admission	665 (17.6)	120 (18.1)	545 (17.5)	0.739
Community hospital admissions	614 (12.2)	101 (16.8)	513 (11.6)	< 0.001
Tertiary hospital admissions	1,648 (11.9)	214 (14.7)	1,434 (11.6)	< 0.001
ICU LOS in days, median (IQR)	4 (2-8)	4 (2-9)	4 (2-8)	0.181
Hospital LOS in days, median (IQR)	15 (8-31)	15 (8-32)	15 (8-31)	0.823

ED, emergency department; ICU, intensive care unit; IQR, interquartile range; LOS, length of stay.

After covariate adjustment, round-time admission remained associated with higher odds of ICU death (OR 1.19, 95% CI 1.03 to 1.38, *P *= 0.017; AUC ROC 0.818, 95% CI 0.809 to 0.827, GoF, *P *= 0.260) but was not significantly associated with hospital mortality (OR 1.02, 95% CI 0.90 to 1.16, *P *= 0.700; AUC ROC 0.798, 95% CI 0.791 to 0.806, GoF, *P *= 0.996) (Tables 4 and 5). There was variability in the adjusted OR for ICU mortality when stratified by study year, and a significantly lower OR for death was observed during the period of 2006-2007 (OR 0.85, 95% CI 0.74 to 0.99, *P *= 0.035).

**Table 4:** Univariate and multi-variable logistic regression analysis showing the association of ICU death with round-time/non-round-time admission.

	Univariate regression			Multi-variable regression		
Predictor variables	OR	95% CI	*P *value	OR	95% CI	*P *value
Admission time						
Non-round time	1.0			1.0		
Round time	1.38	1.21-1.57	< 0.001	1.19	1.03-1.38	0.017
APACHE II score	1.16	1.15-1.16	< 0.001	1.14	1.13-1.15	< 0.001
Age (per year)	1.02	1.02-1.02	< 0.001	1.01	1.00-1.01	< 0.001
Burden of comorbidities						
No comorbidity	1.0			1.0		
One comorbidity	1.87	1.70-2.06	< 0.001	0.98	0.87-1.10	0.752
Two or more comorbidities	2.42	1.97-2.97	< 0.001	1.14	0.90-1.44	0.282
Mechanical ventilation						
No	1.0			1.0		
Yes	3.26	2.79-3.83	< 0.001	1.75	1.47-2.08	< 0.001
Source of admission						
Operating room - Elective	1.0			1.0		
Operating room - Emergency	3.66	2.73-4.91	< 0.001	2.45	1.81-3.34	< 0.001
Emergency department	5.84	4.44-7.69	< 0.001	3.87	2.88-5.20	< 0.001
Other hospital	5.74	4.31-7.62	< 0.001	3.25	2.39-4-42	< 0.001
Ward	8.27	6.26-10.92	< 0.001	3.95	2.92-5.33	< 0.001
Study year						
2002/2003	1.0			1.0		
2004/2005	1.06	0.93-1.21	0.392	0.95	0.82-1.10	0.531
2006/2007	0.93	0.81-1.06	0.285	0.85	0.74-0.99	0.035
2008/2009	1.04	0.91-1.18	0.540	0.93	0.81-1.08	0.356
Study site						
Community hospitals	1.0			1.0		
Tertiary hospitals	0.97	0.87-1.07	0.541	1.00	0.90-1.13	0.925
Admission diagnosis						
Respiratory	1.0			1.0		
Gastrointestinal	1.11	0.97-126	0.120	1.40	1.19-1.64	< 0.001
Cardiovascular	1.53	1.34-1.74	< 0.001	1.81	1.55-2.11	< 0.001
Sepsis	1.57	1.36-1.82	< 0.001	0.91	0.77-1.07	0.251
Trauma	0.42	0.34-0.53	< 0.001	0.87	0.68-1.12	0.289
Metabolic	0.31	0.23-0.41	< 0.001	0.31	0.22-0.41	< 0.001
Neurologic	0.93	0.77-1.12	0.458	1.21	0.99-1.49	0.067
Renal	0.64	0.46-0.88	0.006	0.49	0.34-0.69	< 0.001
Other	0.65	0.46-0.91	0.013	1.06	0.72-1.56	0.760

Area under the receiver operating characteristic curve = 0.818, 95% confidence interval (CI) = 0.809 to 0.827, goodness-of-fit test: 0.260. APACHE II, Acute Physiology and Chronic Health Evaluation II; OR, odds ratio.

**Table 5:** Univariate and multi-variable logistic regression analysis showing the association of hospital death with round-time/non-round-time admission.

	Univariate regression			Multi-variable regression		
Predictor variables	OR	95% CI	*P *value	OR	95% CI	*P *value
Admission time						
Non-round time	1.0			1.0		
Round time	1.21	1.09-1.35	< 0.001	1.02	0.90-1.16	0.700
APACHE II score	1.14	1.13-1.14	< 0.001	1.12	1.11-1.12	< 0.001
Age (per year)	1.03	1.03-1.03	< 0.001	1.02	1.02-1.02	< 0.001
Burden of comorbidities						
No comorbidity	1.0			1.0		
One comorbidity	2.00	1.85-2.17	< 0.001	1.17	1.07-1.29	0.001
Two or more comorbidities	2.48	2.08-2.96	< 0.001	1.31	1.07-1.60	0.008
Mechanical ventilation						
No	1.0			1.0		
Yes	2.11	1.90-2.34	< 0.001	1.42	1.26-1.61	< 0.001
Source of admission						
Operating room - Elective	1.0			1.0		
Operating room - Emergency	2.37	1.98-2.83	< 0.001	1.81	1.49-2.20	< 0.001
Emergency department	3.23	2.75-3.82	< 0.001	2.92	2.42-3.52	< 0.001
Other hospital	2.64	2.21-3.16	< 0.001	2.05	1.68-2.51	< 0.001
Ward	5.59	4.72-6.61	< 0.001	3.72	3.07-4.50	< 0.001
Study year						
2002/2003	1.0			1.0		
2004/2005	1.04	0.94-1.16	0.450	0.98	0.87-1.11	0.778
2006/2007	1.02	0.92-1.14	0.635	1.03	0.91-1.16	0.641
2008/2009	1.07	0.96-1.19	0.211	1.04	0.92-1.17	0.514
Study site						
Community hospitals	1.0			1.0		
Tertiary hospitals	1.00	0.92-1.08	0.966	1.05	0.96-1.15	0.298
Admission diagnosis						
Respiratory	1.0			1.0		
Gastrointestinal	1.27	1.14-1.40	< 0.001	1.70	1.49-1.93	< 0.001
Cardiovascular	1.35	1.21-1.51	< 0.001	1.66	1.46-1.89	< 0.001
Sepsis	1.54	1.37-1.74	< 0.001	1.02	0.89-1.18	0.728
Trauma	0.35	0.29-0.42	< 0.001	0.87	0.71-1.07	0.199
Metabolic	0.29	0.23-0.36	< 0.001	0.38	0.30-0.48	< 0.001
Neurologic	1.15	0.99-1.33	0.053	1.72	1.46-2.02	< 0.001
Renal	0.89	0.71-1.11	0.294	0.71	0.55-0.91	0.007
Other	0.81	0.64-1.03	0.093	1.51	1.13-2.00	0.005

Area under the receiver operating characteristic curve = 0.798, 95% confidence interval (CI) = 0.791 to 0.806, goodness-of-fit test: 0.996. APACHE II, Acute Physiology and Chronic Health Evaluation II; OR, odds ratio.

### Sensitivity analysis

In a further exploratory analysis, higher adjusted ICU mortality for round-time compared with non-round-time admissions was seen among the community ICU (OR 1.43, 95% CI 1.09 to 1.89, *P *= 0.009) when compared with the tertiary ICU subgroup (Additional file 1). However, there remained no significant association between morning round admission and hospital mortality stratified by community or tertiary subgroups (Additional file 2). The subgroup referred from the ED for ICU admission showed a higher adjusted OR for ICU death (OR 1.54, 95% CI 1.21 to 1.95, *P *< 0.001) and a trend toward higher hospital death (OR 1.22, 95% CI 0.99 to 1.51, *P *= 0.057) for round-time admission compared with non-round-time admission (Additional file 3). The subgroup admitted during the years 2006/2007 showed a higher adjusted OR for ICU (OR 1.47, 95% CI 1.11 to 1.95, *P *= 0.008) and hospital death (OR 1.30, 95% CI 1.03 to 1.64, *P *= 0.028) relative to other years for round-time compared with non-round-time admission (Additional file 4).

## Discussion

We performed a large, multi-center, retrospective cohort study to evaluate the association between admission to the ICU during morning bedside rounds and mortality.

### Summary of major findings

We found that approximately 1 in 10 ICU admissions occurs during the period of morning bedside clinical rounds. We found that round-time admissions more commonly occurred in community compared with tertiary hospital ICUs. We also found that patients admitted during round time had higher likelihoods to be transferred from the ward and to be medical (non-operative) and were characterized by a higher burden of comorbid illness and greater illness severity. Likewise, we found that the distribution of admission diagnoses differed; patients arriving during morning rounds were more likely to have respiratory failure and septic diagnoses. Although we showed that patients admitted during morning rounds had higher crude ICU and hospital mortality, the association with ICU mortality remained after adjustment in multi-variable analysis; however, there was no significant association with hospital mortality. Finally, in additional sensitivity analyses, we found a higher mortality associated with admissions occurring during morning rounds for those occurring in community hospitals and for patients referred from the ED.

### Comparison with previous studies

A number of observational studies have evaluated the association between time of ICU admission and hospital mortality and have shown inconsistent findings [11-17]. In data from a large Canadian health region, ICU admission 'after hours' was independently predictive of higher in-hospital mortality [11]. Alternatively, a recent systematic review suggested that weekend, but not night-time, ICU admissions were associated with an increased risk for death [18], implying that differences in ICU organizational structure on weekends and 'after hours' may negatively impact patient outcomes. In a recent French multi-center study, ICU admissions during the weekend or during 'after hours' were not associated with an increased risk of death, whereas ICU discharge 'after hours' independently predicted a worse clinical outcome [19].

To date, only one large retrospective study has investigated an association between ICU admission during morning rounds and mortality: Afessa and colleagues [8] examined 49,844 unique admissions to a single tertiary/academic center encompassing four closed ICUs (two surgical, one medical, and one multi-disciplinary) over the course of a 13-year period and used prospectively collected clinical/administrative data from a local quality/outcomes database. In that study, round-time admission was more narrowly defined (in comparison with our study) as occurring between 8 and 10:59 a.m.; patients admitted 2 hours before or after this time were excluded. Round-time admissions accounted for 7.2% of all ICU admissions; given that we used a slightly more liberal definition, this figure is similar to the 10.9% observed in our study. Patients admitted during round time were similarly characterized by higher illness severity and were more likely to be non-operative in comparison with non-round-time admissions. In that study, round-time admission was associated with a significantly higher hospital mortality (16.2% versus 8.8%, adjusted OR 1.32, 95% CI 1.18 to 1.48, *P *< 0.001). The investigators were also able to show significant declines in mortality for round-time admissions over the course of the study (21.3% in 1994/1995 to 13.6% in 2006/2007) and an association with modifications to the ICU organizational model (change from one- to two-intensivist team, *P *< 0.001; in-house intensivist coverage, *P *< 0.001; introduction of a rapid response team, *P *< 0.001; however, all of these changes occurred very late in the study).

We believe that our data extend the findings of this study, in particular by enabling the evaluation of multiple hospital ICUs, including community-based teaching hospitals, and more extensive covariate adjustment. Our data suggest that ICU admission during morning rounds portends an increased risk for ICU death, and while no statistical difference for in-hospital mortality, certainly fails to suggest that admission during this time leads to improved clinical outcomes, when theoretically all available staff (intensivists, trainees, full complement of multi-disciplinary team members) are present in the ICU.

We believe that the observed differences between community and tertiary hospital ICUs with respect to morning round-time admissions are novel. We observed the proportion of total ICU admissions during round time to be higher in the community as compared with those of tertiary hospital ICUs (12.0% versus 10.5%, *P *= 0.004). This was furthermore associated with an adjusted ICU mortality that was 44% higher and a non-significant increase in hospital mortality, despite comparable patient characteristics and illness severity.

In addition, we found that ICU admission from the ED during morning rounds was associated with greater ICU mortality and a trend for greater hospital mortality. This generates speculation about the influence of system-level factors associated with delays in transfer of critically ill patients from the ED, such as ICU capacity/bed availability, organizational structure, and/or personnel/staffing models, whereby selected patients may be 'held' in the ED during 'after hours' until an ICU bed is available the following morning. In a large observational study using the Project IMPACT database, Chalfin and colleagues [7] found that 2.1% of critically ill patients admitted from the ED to the ICU experienced delays in transfer, defined as a stay in the ED of more than 6 hours. Whereas patients with delayed ED transfer to the ICU had characteristics similar to those of patients without a delayed transfer, the former had a longer median hospital stay (7.0 versus 6.0 days, *P *< 0.001) and greater hospital mortality (17.4% versus 12.9%, *P *< 0.001).

### Study limitations and strengths

Our study has several limitations. First, although our study is large and multi-centric and represents all ICUs in a large Canadian health region, it is a retrospective analysis of prospectively collected data and is observational and thus is potentially predisposed to bias and residual confounding. However, the evaluation of health services, such as how the timing of admission to the ICU impacts patient course and outcomes, cannot feasibly be evaluated in a clinical trial setting. Accordingly, we believe that there is value in replicating observational studies of this nature to assess for consistency and generalizability across studies. Second, despite our adjustment of those variables available, we recognize that we may have omitted additional variables of prognostic importance. Third, we estimated that the routine time during which morning bedside rounds occur at all sites was between 8 and 11:59 a.m. and further recognize that this may have varied at times between and within sites during the study period and depended on additional unrecorded intangible factors.

### Areas of future research

We believe that these observations suggest that system-level factors (such as the relative timing of ICU admission and organizational structure) in addition to patient-level factors (such as comorbidity, illness severity, and admission diagnosis) can have an important modifying effect on the outcome for critically ill patients. However, although our study adds to our current understanding of these patient-system interactions, further confirmatory studies that focus on the relative timing of admission in community/metropolitan hospitals, the impact of delayed transfers from the ED to the ICU, and their interaction with ICU organizational structure and capacity are needed.

## Conclusions

In summary, patients admitted during morning rounds have important differences in baseline characteristics, disposition, and illness severity in comparison with those admitted during other periods of the day. Moreover, in comparison with the latter, the former have a significantly higher ICU mortality but have similar hospital mortality. Importantly, ICU admission during morning rounds was associated with higher mortality in community hospitals and in patients referred from the ED. These data argue for a need to critically evaluate the patient-system factors associated with ICU admission, such as those that occur during morning bedside rounds and that have the potential to impact patient outcome.

## Key messages

• Approximately 1 in 10 intensive care unit (ICU) admissions occurs during morning bedside clinical rounds.

• Patients admitted during morning rounds had higher likelihoods to be transferred from the ward and to be medical (non-operative) and were characterized by a higher burden of comorbid illness and greater illness severity.

• Patients admitted during morning rounds had higher crude and adjusted ICU mortality.

• There was no significant association between morning rounds and hospital mortality after adjustment in multi-variable analysis.

• We found a higher mortality associated with admissions occurring during morning rounds for those occurring in community hospitals and for patients referred from the emergency department.

## Abbreviations

APACHE II: Acute Physiology and Chronic Health Evaluation II; AUR ROC: area under the receiver operating characteristic curve; CI: confidence interval; ED: emergency department; GNH: Grey Nuns Community Hospital; GoF: goodness-of-fit; ICU: intensive care unit; OR: odds ratio; RAH: Royal Alexandria Hospital; UAH: University of Alberta Hospital.

## Competing interests

The authors declare that they have no competing interests.

## Authors' contributions

IS participated in the design of the study, performed statistical analysis, and drafted the manuscript. CK and RG participated in the design of the study and helped draft the manuscript. SB conceived the study, participated in its design and coordination, performed statistical analysis, and helped to draft the manuscript. All authors read and approved the final manuscript.

## Supplementary Material

Additional file 1 **Sensitivity Analysis**. Multiple variable logistic regression analysis showing the association of ICU death with round-time/non-round-time admission, APACHE II score, age, burden of comorbidities, mechanical ventilation at admission, source of admission, study year and admission diagnosis stratified by study site. Click here for file

Additional file 2 **Sensitivity Analysis**. Multiple variable logistic regression analysis showing the association of hospital death with round-time/non-round-time admission, APACHE II score, age, burden of comorbidities, mechanical ventilation at admission, source of admission, study year and admission diagnosis stratified by study site. Click here for file

Additional file 3 **Sensitivity Analysis**. Multiple variable logistic regression analysis showing the association of ICU and hospital death with round-time/non-round-time admission, APACHE II score, age, burden of comorbidities, mechanical ventilation at admission, study year, study site and admission diagnosis among patients referred from the Emergency Department. Click here for file

Additional file 4 **Sensitivity Analysis**. Multiple variable logistic regression analysis showing the association of ICU and hospital death with round-time/non-round-time admission, APACHE II score, age, burden of comorbidities, mechanical ventilation at admission, source of admission, study site and admission diagnosis among patients admitted during years 2006/2007. Click here for file
