# Supplementary material for: Impact of intensive care unit admission during morning bedside rounds and mortality: a multi-center retrospective cohort study
Source: Crit Care. 2012 May 3;16(3):R72. doi: 10.1186/cc11329 (PMC3580614; doi:10.1186/cc11329)
Supplement: Additional file 1 — Sensitivity Analysis. Multiple variable logistic regression analysis showing the association of ICU death with round-time/non-round-time admission, APACHE II score, age, burden of comorbidities, mechanical ventilation at admission, source of admission, study year and admission diagnosis stratified by study site. [file cc11329-S1.DOC]

**Additional File 1 -** Multiple variable logistic regression analysis showing the association of ICU death with round-time/non-round-time admission, APACHE II score, age, burden of comorbidities, mechanical ventilation at admission, source of admission, study year and admission diagnosis stratified by study site.

|  | **Community Hospitals Subgroup** | | | **Tertiary Hospitals Subgroup** | | |
| --- | --- | --- | --- | --- | --- | --- |
| **Predictor Variables** | **OR** | **95% CI** | **p-value** | **OR** | **95% CI** | **p-value** |
| **Admission Time** |  |  |  |  |  |  |
| Non-Round-time | 1.0 |  |  | 1.0 |  |  |
| Round-time | 1.43 | 1.09-1.89 | 0.009 | 1.11 | 0.93-1.32 | 0.243 |
| **APACHE II score** | 1.14 | 1.13-1.16 | <0.001 | 1.14 | 1.13-1.15 | <0.001 |
| **Age (per year)** | 1.01 | 1.01- 1.02 | <0.001 | 1.00 | 1.00-1.01 | 0.040 |
| **Burden of Comorbidities** |  |  |  |  |  |  |
| No comorbidity | 1.0 |  |  | 1.0 |  |  |
| Just one comorbidity | 0.99 | 0.78-1.26 | 0.958 | 0.98 | 0.85-1.11 | 0.717 |
| Two or more comorbidities | 0.77 | 0.45-1.31 | 0.339 | 1.24 | 0.95-1.61 | 0.107 |
| **Mechanical Ventilation** |  |  |  |  |  |  |
| No | 1.0 |  |  | 1.0 |  |  |
| Yes | 2.33 | 1.65-3.30 | <0.001 | 1.55 | 1.26-1.89 | <0.001 |
| **Source of admission** |  |  |  |  |  |  |
| Operating room - Elective | 1.0 |  |  | 1.0 |  |  |
| Operating room – Emergency | 3.09 | 1.70-5.60 | <0.001 | 2.17 | 1.51-3.10 | <0.001 |
| Emergency department | 5.11 | 2.86-9.13 | <0.001 | 3.41 | 2.42-4.82 | <0.001 |
| Other hospital | 5.25 | 2.91-9.42 | <0.001 | 2.61 | 1.81-3.75 | <0.001 |
| Ward | 5.22 | 2.90-9.42 | <0.001 | 3.47 | 2.44-4.92 | <0.001 |
| **Study year** |  |  |  |  |  |  |
| 2002/2003 | 1.0 |  |  | 1.0 |  |  |
| 2004/2005 | 0.95 | 0.70-1.31 | 0.776 | 0.96 | 0.81-1.13 | 0.621 |
| 2006/2007 | 1.01 | 0.75-1.36 | 0.956 | 0.81 | 0.68-0.96 | 0.016 |
| 2008/2009 | 1.09 | 0.82-1.45 | 0.560 | 0.89 | 0.76-1.06 | 0.196 |
| **Admission diagnosis** |  |  |  |  |  |  |
| Respiratory | 1.0 |  |  | 1.0 |  |  |
| Gastrointestinal | 1.47 | 1.05-2.06 | 0.023 | 1.39 | 1.15-1.66 | <0.001 |
| Cardiovascular | 1.89 | 1.43-2.51 | <0.001 | 1.81 | 1.51-2.18 | <0.001 |
| Sepsis | 1.06 | 0.77-1.47 | 0.702 | 0.86 | 0.71-1.05 | 0.131 |
| Trauma | 0.66 | 0.08-5.27 | 0.699 | 0.84 | 0.65-1.10 | 0.207 |
| Metabolic | 0.35 | 0.20-0.62 | <0.001 | 0.30 | 0.21-0.43 | <0.001 |
| Neurologic | 0.77 | 0.42-1.42 | 0.406 | 1.28 | 1.02-1.59 | 0.034 |
| Renal | 0.56 | 0.25-1.21 | 0.140 | 0.47 | 0.32-0.69 | <0.001 |
| Other | 1.21 | 0.50-2.93 | 0.661 | 1.04 | 0.68-1.60 | 0.843 |

Abbreviations: OR = odds ratio; APACHE = Acute Physiology and Chronic Health Evaluation.

Community Hospitals Subgroup: AuROC: 0.841 (95% CI= 0.826-0.856), GoF test: 0.999.

Tertiary Hospitals Subgroup: AuROC: 0.812 (95% CI= 0.801-0.822), GoF test: 0.111.
