# Supplementary material for: Impact of intensive care unit admission during morning bedside rounds and mortality: a multi-center retrospective cohort study
Source: Crit Care. 2012 May 3;16(3):R72. doi: 10.1186/cc11329 (PMC3580614; doi:10.1186/cc11329)
Supplement: Additional file 2 — Sensitivity Analysis. Multiple variable logistic regression analysis showing the association of hospital death with round-time/non-round-time admission, APACHE II score, age, burden of comorbidities, mechanical ventilation at admission, source of admission, study year and admission diagnosis stratified by study site. [file cc11329-S2.DOC]

**Additional File 2 -** Multiple variable logistic regression analysis showing the association of hospital death with round-time/non-round-time admission, APACHE II score, age, burden of comorbidities, mechanical ventilation at admission, source of admission, study year and admission diagnosis stratified by study site.

|  | **Community Hospitals Subgroup** | | | **Tertiary Hospitals Subgroup** | | |
| --- | --- | --- | --- | --- | --- | --- |
| **Predictor Variables** | **OR** | **95% CI** | **p-value** | **OR** | **95% CI** | **p-value** |
| **Admission Time** |  |  |  |  |  |  |
| Non-Round time | 1.0 |  |  | 1.0 |  |  |
| Round-time | 1.13 | 0.90-1.43 | 0.291 | 0.99 | 0.85-1.14 | 0.870 |
| **APACHE II score** | 1.12 | 1.11-1.13 | <0.001 | 1.11 | 1.11-1.13 | <0.001 |
| **Age (per year)** | 1.03 | 1.02-1.03 | <0.001 | 1.02 | 1.02-1.02 | <0.001 |
| **Burden of Comorbidities** |  |  |  |  |  |  |
| No comorbidity | 1.0 |  |  | 1.0 |  |  |
| Just one comorbidity | 1.15 | 0.95-1.40 | 0.152 | 1.17 | 1.05-1.31 | 0.004 |
| Two or more comorbidities | 1.05 | 0.68-1.63 | 0.819 | 1.37 | 1.09-1.72 | 0.006 |
| **Mechanical Ventilation** |  |  |  |  |  |  |
| No | 1.0 |  |  | 1.0 |  |  |
| Yes | 1.73 | 1.37-2.18 | <0.001 | 1.30 | 1.12-1.50 | <0.001 |
| **Source of admission** |  |  |  |  |  |  |
| Operating room - Elective | 1.0 |  |  | 1.0 |  |  |
| Operating room – Emergency | 1.84 | 1.27-2.66 | 0.001 | 1.73 | 1.38-2.18 | <0.001 |
| Emergency department | 3.08 | 2.15-4.42 | <0.001 | 2.76 | 2.21-3.44 | <0.001 |
| Other hospital | 2.89 | 1.98-4.21 | <0.001 | 1.67 | 1.32-2.14 | <0.001 |
| Ward | 4.01 | 2.78-5.81 | <0.001 | 3.47 | 2.77-4.34 | <0.001 |
| **Study year** |  |  |  |  |  |  |
| 2002/2003 | 1.0 |  |  | 1.0 |  |  |
| 2004/2005 | 1.09 | 0.84-1.41 | 0.505 | 0.95 | 0.83-1.09 | 0.512 |
| 2006/2007 | 1.21 | 0.94-1.54 | 0.133 | 0.98 | 0.86-1.13 | 0.827 |
| 2008/2009 | 1.33 | 1.05-1.68 | 0.019 | 0.96 | 0.84-1.10 | 0.552 |
| **Admission diagnosis** |  |  |  |  |  |  |
| Respiratory | 1.0 |  |  | 1.0 |  |  |
| Gastrointestinal | 1.70 | 1.29-2.25 | <0.001 | 1.71 | 1.48-1.99 | <0.001 |
| Cardiovascular | 1.50 | 1.18-1.91 | 0.001 | 1.81 | 1.55-2.12 | <0.001 |
| Sepsis | 1.11 | 0.84-1.45 | 0.462 | 1.01 | 0.86-1.19 | 0.926 |
| Trauma | 1.09 | 0.28-4.19 | 0.901 | 0.86 | 0.69-1.06 | 0.160 |
| Metabolic | 0.35 | 0.22-0.55 | <0.001 | 0.39 | 0.30-0.52 | <0.001 |
| Neurologic | 1.87 | 1.23-2.86 | 0.004 | 1.68 | 1.41-2.01 | <0.001 |
| Renal | 0.58 | 0.32-1.07 | 0.083 | 0.74 | 0.57-0.98 | 0.035 |
| Other | 1.81 | 0.98-3.32 | 0.055 | 1.45 | 1.05-2.00 | 0.023 |

Abbreviations: OR = odds ratio; APACHE = Acute Physiology and Chronic Health Evaluation.

Community Hospitals Subgroup: AuROC: 0.811 (95% CI= 0.798-0.825), GoF test: 0.960.

Tertiary Hospitals Subgroup: AuROC: 0.795 (95% CI= 0.787-0.804), GoF test: 0.984.
