# Supplementary material for: Impact of intensive care unit admission during morning bedside rounds and mortality: a multi-center retrospective cohort study
Source: Crit Care. 2012 May 3;16(3):R72. doi: 10.1186/cc11329 (PMC3580614; doi:10.1186/cc11329)
Supplement: Additional file 3 — Sensitivity Analysis. Multiple variable logistic regression analysis showing the association of ICU and hospital death with round-time/non-round-time admission, APACHE II score, age, burden of comorbidities, mechanical ventilation at admission, study year, study site and admission diagnosis among patients referred from the Emergency Department. [file cc11329-S3.DOC]

**Additional File 3 - Multiple variable logistic regression analysis showing the association of ICU and hospital death with round-time/non-round-time admission, APACHE II score, age, burden of comorbidities, mechanical ventilation at admission, study year, study site and admission diagnosis among patients referred from the Emergency Department.**

|  | **ICU death** | | | **Hospital Death** | | |
| --- | --- | --- | --- | --- | --- | --- |
| **Predictor Variables** | **OR** | **95% CI** | **p-value** | **OR** | **95% CI** | **p-value** |
| **Admission Time** |  |  |  |  |  |  |
| Non-Round time | 1.0 |  |  | 1.0 |  |  |
| Round-time | 1.54 | 1.21-1.95 | <0.001 | 1.22 | 0.99-1.51 | 0.057 |
| **APACHE II score** | 1.16 | 1.14-1.17 | <0.001 | 1.13 | 1.12-1.15 | <0.001 |
| **Age (per year)** | 1.00 | 0.99-1.01 | 0.093 | 1.02 | 1.02-1.02 | <0.001 |
| **Burden of Comorbidities** |  |  |  |  |  |  |
| No comorbidity | 1.0 |  |  | 1.0 |  |  |
| Just one comorbidity | 0.83 | 0.68-1.01 | 0.058 | 1.07 | 0.91-1.26 | 0.378 |
| Two or more comorbidities | 0.99 | 0.66-1.48 | 0.953 | 1.25 | 0.88-1.77 | 0.208 |
| **Mechanical Ventilation** |  |  |  |  |  |  |
| No | 1.0 |  |  | 1.0 |  |  |
| Yes | 1.51 | 1.14-2.00 | 0.004 | 1.27 | 1.04-1.56 | 0.019 |
| **Study year** |  |  |  |  |  |  |
| 2002/2003 | 1.0 |  |  | 1.0 |  |  |
| 2004/2005 | 0.77 | 0.60-0.99 | 0.039 | 0.87 | 0.71-1.08 | 0.209 |
| 2006/2007 | 0.75 | 0.59-0.96 | 0.021 | 0.92 | 0.75-1.12 | 0.415 |
| 2008/2009 | 0.81 | 0.64-1.03 | 0.086 | 0.99 | 0.81-1.20 | 0.903 |
| **Study site** |  |  |  |  |  |  |
| Community Hospitals | 1.0 |  |  | 1.0 |  |  |
| Tertiary Hospitals | 1.09 | 0.90-1.33 | 0.372 | 1.18 | 1.00-1.39 | 0.043 |
| **Admission diagnosis** |  |  |  |  |  |  |
| Respiratory | 1.0 |  |  | 1.0 |  |  |
| Gastrointestinal | 1.36 | 1.02-1.83 | 0.039 | 1.49 | 1.16-1.91 | 0.002 |
| Cardiovascular | 2.25 | 1.75-2.89 | <0.001 | 2.23 | 1.78-2.78 | <0.001 |
| Sepsis | 0.89 | 0.69-1.16 | 0.414 | 0.91 | 0.73-1.14 | 0.418 |
| Trauma | 1.00 | 0.71-1.41 | 0.996 | 0.89 | 0.67-1.19 | 0.442 |
| Metabolic | 0.33 | 0.22-0.49 | <0.001 | 0.37 | 0.28-0.51 | <0.001 |
| Neurologic | 1.57 | 1.18-2.08 | 0.002 | 1.91 | 1.52-2.41 | <0.001 |
| Renal | 0.23 | 0.11-0.47 | <0.001 | 0.44 | 0.28-0.69 | <0.001 |
| Other | 0.93 | 0.34-2.53 | 0.890 | 1.09 | 0.47-2.51 | 0.843 |

Abbreviations: OR = odds ratio; APACHE = Acute Physiology and Chronic Health Evaluation.

Dependent variable: ICU Death; AuROC: 0.814(95% CI= 0.799-0.829), GoF test: 0.255.

Dependent variable: Hospital Death; AuROC: 0.804 (95% CI= 0.791-0.816), GoF test: 0.976.
