# Supplementary material for: Impact of intensive care unit admission during morning bedside rounds and mortality: a multi-center retrospective cohort study
Source: Crit Care. 2012 May 3;16(3):R72. doi: 10.1186/cc11329 (PMC3580614; doi:10.1186/cc11329)
Supplement: Additional file 4 — Sensitivity Analysis. Multiple variable logistic regression analysis showing the association of ICU and hospital death with round-time/non-round-time admission, APACHE II score, age, burden of comorbidities, mechanical ventilation at admission, source of admission, study site and admission diagnosis among patients admitted during years 2006/2007. [file cc11329-S4.DOC]

**Additional File 4 -** Multiple variable logistic regression analysis showing the association of ICU and hospital death with round-time/non-round-time admission, APACHE II score, age, burden of comorbidities, mechanical ventilation at admission, source of admission, study site and admission diagnosis among patients admitted during years 2006/2007.

|  | **ICU death** | | | **Hospital Death** | | |
| --- | --- | --- | --- | --- | --- | --- |
| **Predictor Variables** | **OR** | **95% CI** | **p-value** | **OR** | **95% CI** | **p-value** |
| **Admission Time** |  |  |  |  |  |  |
| Non-Round time | 1.0 |  |  | 1.0 |  |  |
| Round-time | 1.47 | 1.11-1.95 | 0.008 | 1.30 | 1.03-1.64 | 0.028 |
| **APACHE II score** | 1.15 | 1.13-1.16 | <0.001 | 1.12 | 1.10-1.13 | <0.001 |
| **Age (per year)** | 1.01 | 1.00-1.02 | 0.004 | 1.03 | 1.02-1.03 | <0.001 |
| **Burden of Comorbidities** |  |  |  |  |  |  |
| No comorbidity | 1.0 |  |  | 1.0 |  |  |
| Just one comorbidity | 0.86 | 0.68-1.09 | 0.217 | 1.02 | 0.85-1.24 | 0.808 |
| Two or more comorbidities | 1.31 | 0.80-2.12 | 0.279 | 1.51 | 1.01-2.27 | 0.045 |
| **Mechanical Ventilation** |  |  |  |  |  |  |
| No | 1.0 |  |  | 1.0 |  |  |
| Yes | 1.66 | 1.16-2.37 | 0.005 | 1.40 | 1.11-1.78 | 0.005 |
| **Source of admission** |  |  |  |  |  |  |
| Operating room - Elective | 1.0 |  |  | 1.0 |  |  |
| Operating room – Emergency | 1.95 | 1.08-3.51 | 0.026 | 1.66 | 1.15-2.40 | 0.007 |
| Emergency department | 3.48 | 1.98-6.12 | <0.001 | 2.52 | 1.76-3.61 | <0.001 |
| Other hospital | 2.46 | 1.36-4.46 | 0.003 | 1.61 | 1.09-2.39 | 0.016 |
| Ward | 3.55 | 2.00-6.29 | <0.001 | 3.48 | 2.41-5.02 | <0.001 |
| **Study site** |  |  |  |  |  |  |
| Community Hospitals | 1.0 |  |  | 1.0 |  |  |
| Tertiary Hospitals | 0.94 | 0.75-1.18 | 0.613 | 1.08 | 0.90-1.29 | 0.415 |
| **Admission diagnosis** |  |  |  |  |  |  |
| Respiratory | 1.0 |  |  | 1.0 |  |  |
| Gastrointestinal | 1.74 | 1.27-2.38 | 0.001 | 1.97 | 1.53-2.54 | <0.001 |
| Cardiovascular | 1.99 | 1.47-2.69 | <0.001 | 1.68 | 1.30-2.17 | <0.001 |
| Sepsis | 0.93 | 0.67-1.28 | 0.643 | 1.14 | 0.88-1.48 | 0.325 |
| Trauma | 0.59 | 0.33-1.06 | 0.080 | 0.75 | 0.50-1.14 | 0.185 |
| Metabolic | 0.29 | 0.15-0.55 | <0.001 | 0.43 | 0.27-0.68 | <0.001 |
| Neurologic | 1.15 | 0.75-1.76 | 0.526 | 1.76 | 1.28-2.44 | 0.001 |
| Renal | 0.42 | 0.21-0.84 | 0.014 | 0.67 | 0.42-1.06 | 0.090 |
| Other | 1.39 | 0.63-3.08 | 0.410 | 1.47 | 0.79-2.74 | 0.220 |

Abbreviations: OR = odds ratio; APACHE = Acute Physiology and Chronic Health Evaluation.

Dependent variable: ICU Death; AuROC: 0.830(95% CI= 0.812-0.847), GoF test: 0.932.

Dependent variable: Hospital Death; AuROC: 0.806 (95% CI= 0.792-0.820), GoF test: 0.991.
